# Effects of Electric Field on Chemical Looping Combustion: A DFT Study of CO Oxidation on CuO (111) Surface

**DOI:** 10.1021/acsomega.4c00743

**Published:** 2024-05-01

**Authors:** Zhongze Bai, Xi Zhuo Jiang, Kai H. Luo

**Affiliations:** † Department of Mechanical Engineering, University College London, Torrington Place, London WC1E 7JE, U.K.; ‡ School of Mechanical Engineering and Automation, Northeastern University, Shenyang, Liaoning 110819, P. R. China

## Abstract

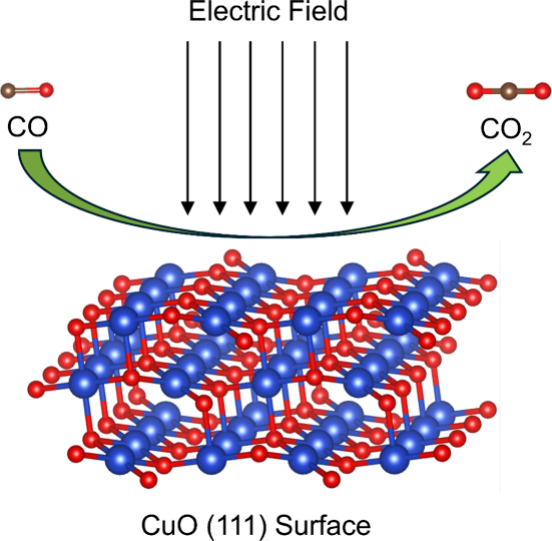

Chemical looping combustion (CLC) is a promising and novel technology for carbon dioxide (CO_2_) capture with a relatively low energy consumption and cost. CuO, one of the most attractive oxygen carriers (OCs) for carbon dioxide (CO) oxidation, suffers from sintering and agglomeration during the reduction process. Applying an electric field (EF) may promote the CO oxidation process on the CuO surface, which could mitigate sintering and agglomeration by decreasing operating temperatures with negligible combustion efficiency loss. This study performs density functional theory (DFT) simulations to investigate the effects of EF on the oxidation of CO on the CuO (111) surface. The results indicate that both the orientation and strength of the EF can significantly affect the oxidation characteristics of CO on the CuO (111) surface such as total reaction energy, energy barriers of reactions, CO adsorption, and CO_2_ desorption. For the first time, this study reveals the role of EF in enhancing CO oxidation through CLC processes via first-principle calculations. Such findings could provide new strategies to improve the performance of CLC processes.

## 1 Introduction

Chemical looping combustion (CLC) is regarded as a promising and novel technology for CO_2_ capture during fuel combustion with relatively low energy consumption and cost, which could help address global warming issues.^1−4^ During such a process, the oxygen carrier (OC) is used to transfer oxygen for fuel combustion, which could avoid the direct contact between fuel and air and obtain high purity CO_2_ without the mixture with N_2_. OCs, the key component for fuel combustion performance in the CLC process, have been synthesized diversely, while improvements on reactivity, thermal stability, resistance to agglomeration, and sintering are still needed.^5,6^

Applying an external electric field (EF) to the CLC process could be an effective approach to enhancing the behaviors of OCs. During this process, EF can rearrange the electronic orbitals of intermediates, altering the binding energies and reaction mechanisms.^7^ Therefore, the exploration of EF influence on the CLC process is of great importance. Among the numerous materials, CuO is one of the suitable OCs because of its high reactivity and oxygen transport capacity, suitable equilibrium partial pressure of oxygen under combustion temperature, stable recyclability of oxygen release and uptake; however, CuO suffers from sintering and agglomeration during the reduction process.^6^ There have been many efforts in the past to improve the performance of OCs by reducing CO oxidation temperatures during CLC in synthesizing nanomaterials, alloys, etc.^8−13^ For instance, the CO oxidation on CuO-CeO_2_ catalysts was explored by a series of experiments.^8,10−12^ Varghese and co-workers investigated the CO oxidation on CuO-Co_3_O_4_ catalyst and found that CuO-Co_3_O_4_ catalyst exhibits superior catalytic properties over pure Co_3_O_4_.^9^ Zedan and co-workers improved the reducibility and stability of CuO in the generation of CuO nanoparticles.^13^ In the present work, we chose CO (the main component for carbon-containing fuels) oxidation on the CuO surface as a representation to study the influence of EF on the CLC process. Hopefully, EF would promote CO oxidation process on CuO surface, which can effectively mitigate sintering and agglomeration by decreasing operating temperatures while maintaining the high combustion efficiency of the CLC process.^6^

Recently, Cu-based OC has attracted much attention from researchers. Three types of mechanisms occur in a CLC process, including the Mars–van Krevelen (MvK) mechanism,^14,15^ Eley–Rideal (ER) mechanism^16,17^ and Langmuir–Hinshelwood (LH) mechanism,^16,17^ respectively. Wu and co-workers investigated the reaction mechanisms of CO and O_2_ over the CuO (111) surface through density functional theory (DFT) calculations,^18^ and found that the reactions between CO and lattice O of CuO (111) surface by the MvK mechanism were less active than those between CO and adsorbed oxygen-containing species (O and O_2_). Zheng and co-workers explored the NOx removal behaviors during a CLC process by studying microscopic reactions between HCN heterogeneous reactions on CuO surface by DFT calculations.^19^ The effects of sulfur-containing species (H_2_S, HS and S) on CO oxidation over CuO surface were revealed by Zheng and Zhao through DFT simulations.^20^ Although the CO oxidation mechanisms over CuO surfaces under various conditions were reported in previous studies, the exploration of EF influence on CO combustion over CuO surfaces was rarely seen, and it is worth exploring the effects of EF on a CLC process.

In the present study, the role of EF in the reaction of CO oxidation on CuO surfaces, a widely accepted route for fuels oxidation by metal oxide materials,^21^ is investigated following the MvK mechanism. In such a process, CO is adsorbed on the CuO surfaces and reacts with lattice O forming adsorbed CO_2_; the desorbed CO_2_ detaches from the CuO surfaces in a gas phase and leave an O vacancy subsequently.^18^ The effects of EF on every individual step of CO oxidation over CuO surfaces are explored via DFT calculations in the present study, including the EF influence on the adsorption and desorption processes of CO and CO_2_, and the chemical processes from CO to CO_2_.

## 2 Methods

All DFT calculations were carried out using the Vienna Ab initio Simulation Package (VASP) package^22,23^ with generalized gradient approximation (GGA) and Perdew–Bruke–Ernzerh (PBE).^24^ Plane wave energy cutoff and the convergence criteria for total energy and forces were set to 500 eV, 1.0 × 10^–5^ eV and 0.03 eV/Å, respectively. The DFT-D3 method with Becke–Johnson damping was used to consider van der Waals interaction.^25,26^ GGA + U with the value of 7.5 eV was adopted to consider the strong electron correlations for Cu atoms.^4^

**Figure 1:**
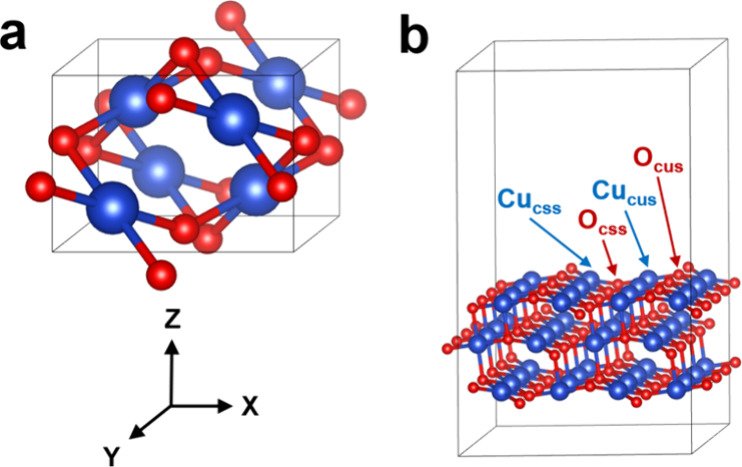
(a) CuO unit cell; (b) three-layer CuO (111) slab. Cu and O atoms are represented in blue and red, respectively.

The selection of CuO unit cell, as shown in Figure 1a, (*a* = 4.631 Å, *b* = 3.418 Å, *c* = 5.079 Å and β = 100.01°) agrees well with experimental parameters (*a* = 4.682 Å, *b* = 3.424 Å, *c* = 5.127 Å and β = 99.42°) with an average error of only 0.4%.^27^ A three-layer P (2 × 2) CuO (111) slab with 15 Å vacuum space, which is the most used model surface because it has the lowest surface energy,^28^ was constructed, as shown in Figure 1b. Four kinds of top sites on the CuO (111) surface were constructed including the saturated 4-fold copper site (Cu_CSS_), the unsaturated 3-fold copper site (Cu_CUS_), the saturated 4-fold oxygen site (O_CSS_) and the unsaturated 3-fold oxygen site (O_CUS_). Monkhorst–Pack k-point grids of 7 × 9 × 6 and 3 × 2 × 1 were used for CuO bulk cell and CuO slab, respectively. To determine reaction barriers, the climbing image nudged elastic band (CI-NEB) method was carried out to search for transition state (TS).^29^ The calculations were assisted by the VASPKIT^30^ and QVASP^31^ code. The influence of EF on CO oxidation on CuO (111) was investigated by imposing EF ranging from −1 to 1 V/Å along the Z axis, where positive and negative values of the EF were imposed along the + Z and -Z directions, respectively.

To facilitate the study, the adsorption energy (*E*_ads_), desorption energy (*E*_des_), energy barrier (*E*_b_) and overall reaction energy (*E*_all_) are calculated as eqs 1–4, respectively.
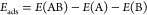
1
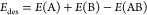
2
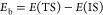
3
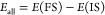
4where, *E*(AB), *E*(A), *E*(B), *E*(TS), *E*(IS) and *E*(FS) are the energies of adsorption structure, substrate, adsorbate, transition state, initial state, and final state, respectively. Lower values of *E*_ads_ and *E*_des_ indicate higher adsorption and desorption abilities of the molecules.

## 3 Results

### 3.1 Effects of Electric Field on CO Adsorption on CuO (111) Surface

**Figure 2:**
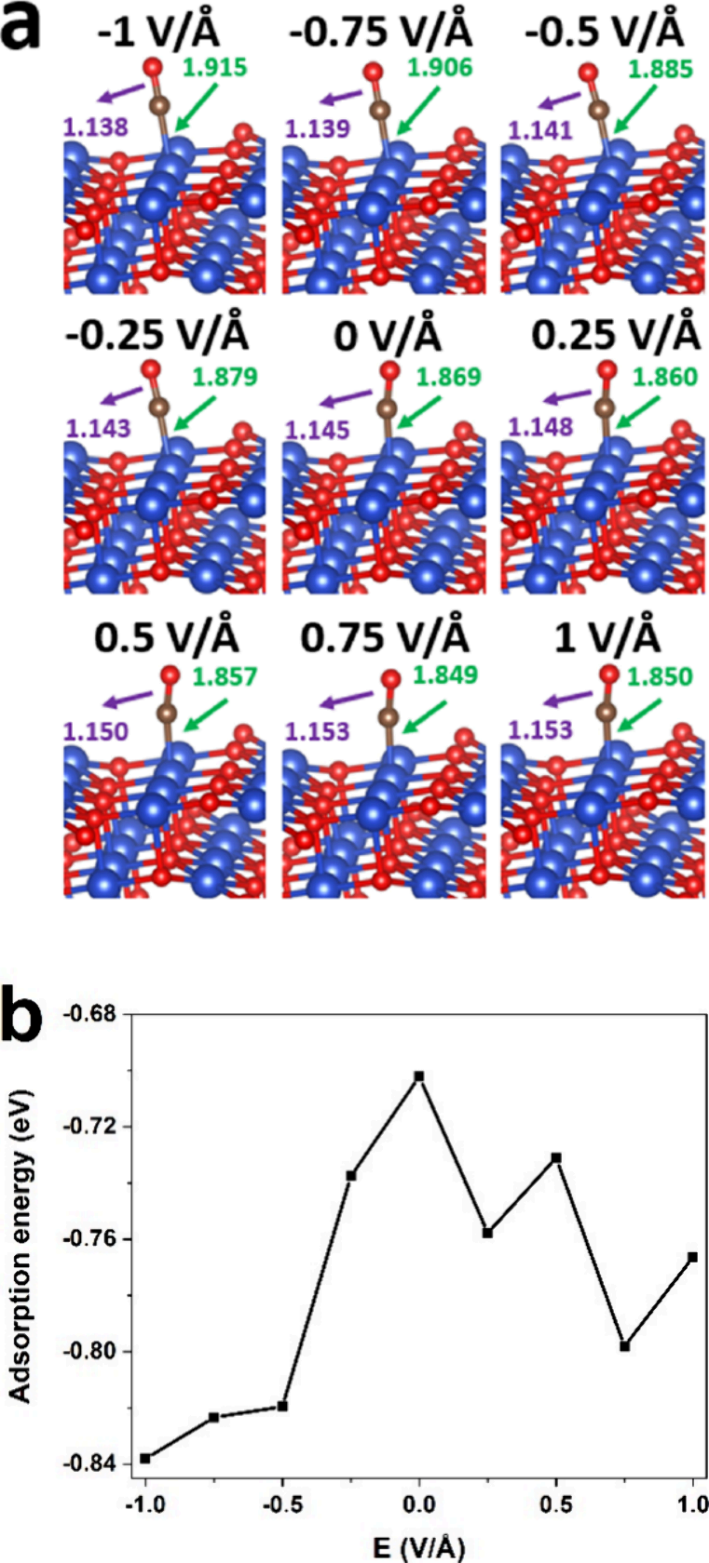
(a) Adsorption structures of CO on the CuO(111) surface under varying EF values. Cu and O atoms are represented in blue and red, respectively. The numbers in figure represent bond length (Å). (b) Effects of EF on adsorption energy of CO on CuO (111) surface.

The most stable adsorption structures of CO on CuO (111) under varying EF strengths are illustrated in Figure 2a after calculation of the adsorption energies of CO at all adsorption sites, including the top, bridge, and hollow sites. Under the EF-free case, Cu_cus_ is the most stable adsorption site for CO, and the bond lengths of C–O as well as Cu–C are 1.145 and 1.869 Å, respectively. Those adsorption parameters are in good agreement with previous simulation results.^18^ Although EF has no effect on the adsorption sites of CO, the direction and magnitude of EF could alter other CO adsorption behaviors significantly. Specifically, the Cu–C and C–O bond lengths decrease and increase, respectively, with *E* increasing from −1 to 0.75 V/Å. In the *E* = 1 V/Å case, the bond length of Cu–C is longer than that in the *E* = 0.75 V/Å case; for the C–O bond, it stays the same in the *E* = 0.75 V/Å conditions. Figure 2b presents the adsorption energy of CO on the CuO (111) surface under varying EF values. When the EF strength ranges from −1 to 0 V/Å, the CO adsorption energy presents an upward trend with *E* rising. When *E* is greater than 0, the adsorption energy of CO decreases in a fluctuating manner with the increase in EF strength.

To further explain EF influence on CO adsorption characteristics at the electronic level on the CuO (111) surface, the Bader charge and partial density of states (PDOS) were analyzed for the adsorption process. Table 1 summarizes the charge transfers of C, Cu and O atoms as well as band centers of Cu and C atoms during CO adsorption on the CuO (111) surface with *E* = −1 to 1 V/Å. For PDOS analysis, the selection of the energy window, −12 to 2 eV, refers to previous work.^18^ For bonding orbitals, 3d and 2p are chosen for Cu and C atoms to characterize the strength of interatomic interactions, respectively.

**Table 1:** Charge Transfers of Cu, O and C Atoms as well as Band Centers of Cu and C Atoms during CO Adsorbs on CuO (111) Surface with *E* Ranging from −1 to 1 V/Å

***E***(V/Å)	**–1**	**–0.75**	**–0.5**	**–0.25**	**0**	**0.25**	**0.5**	**0.75**	**1**
**Charge transfer (|e|)**									
C → Cu	0.120	0.102	0.082	0.062	0.048	0.027	–0.002	–0.024	–0.019
C → O	0.977	0.991	1.035	1.039	1.058	1.064	1.085	1.099	1.096
Total C loss charge	1.097	1.093	1.117	1.101	1.106	1.091	1.083	1.076	1.077
**Bond center (eV)**									
Cu-3d	–3.367	–3.386	–3.441	–3.445	–3.481	–3.509	–3.515	–3.499	–3.519
C-2p	–5.810	–6.013	–6.250	–6.458	–6.495	–6.375	–6.172	–5.033	–5.596

In *E* = −1 to 0.25 V/Å cases, electrons transfer from C to Cu atoms on CuO (111) with CO-adsorbed structures, while electrons will transfer from Cu to C atoms when *E* is higher than 0.25 V/Å. Overall, the increasing EF strength inhibits electron transfer from C to Cu atoms with *E* values of −1 to 0.75 V/Å. When *E* is 1 V/Å, EF promotes electron transfer from the C to Cu atoms. For O atoms, EF enhances electron transfer from C to O atoms until *E* = 0.75 V/Å. The total C loss electrons are less sensitive to the applied external EF, which indicates that the EF alters the electron distribution from Cu atoms to O atoms. During the adsorption process, the O and C atoms are connected by a covalent bond, and the O atom is in saturated status. By contrast, the Cu atoms in Cu_CUS_ are unsaturated. Thus, fewer charge transfers from C to the O and Cu atoms will benefit O and Cu atoms reaching stable states and forming more stable bonds with shorter bond lengths. Based on the above analysis of charge transfers, consequently, the bond lengths of Cu–C and C–O present a downward trend and an upward trend until *E* = 0.75 V/Å.

To better understand the effects of EF on the CO adsorption energy on the CuO(111) surface, we also carried out PDOS analysis as shown in Table 1. The bond centers of Cu-3d decrease from −3.367 eV to −3.519 eV with EF strength ranging from −1 V/Å to 1 V/Å, which means the Cu-3d bond center leaves the Fermi energy level (0 eV) and the interactions of Cu with adsorbates decrease correspondingly. The C-2p bond center decreases with *E* ranging from −1 to 0 V/Å and shows an opposite trend when *E* is higher than 0 V/Å. Both negative and positive EF can enhance the reactivity of C atoms. The absorption energy decreases because the interactions of Cu and C atoms are inhibited by the increasing *E* values when *E* is lower than 0 V/Å. In the *E* = 0 to 1 V/Å conditions, the increase in EF strength promotes bonding of C atoms more than it inhibits bonding of Cu atoms, leading to a decrease in the adsorption energy of CO on the Cu (111) surface.

### 3.2 Effects of Electric Field on CO_2_ Desorption on CuO (111) Surface with O Vacancy

**Figure 3:**
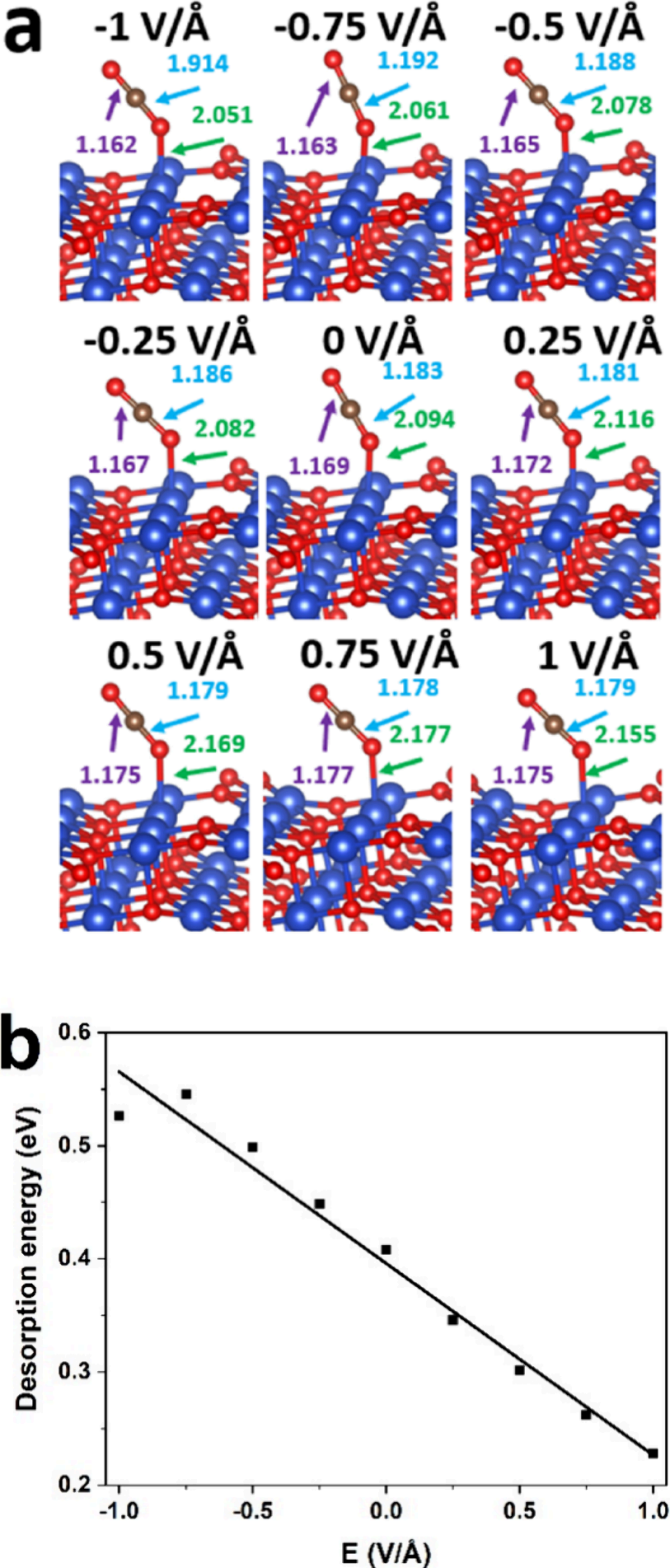
(a) Adsorption structures of CO_2_ on the CuO(111) surface with an O vacancy under varying EF values. Cu and O atoms are represented in blue and red, respectively. The numbers in the figure represent bond length (Å). (b) Effects of EF on the desorption energy of CO_2_ on the CuO(111) surface with an O vacancy.

Subsequently, we explored the CO_2_ adsorption configurations on the CuO (111) surface with an O vacancy under all EF conditions as shown in Figure 3a. The adsorption sites on the CuO surface of CO_2_ are the same as those of CO adsorption and remain unchanged in all cases with and without EF. Different from the CO adsorption configurations, the adsorption sites in CO_2_ are O (lattice) atoms. The bond lengths of C–O and Cu–O (lattice) follow the same trend: the bond lengths increase when *E* ranges from −1 to 0.75 V/Å and decrease in the *E* = 1 V/Å case. The EF influence on C–O (lattice) bond lengths shows an opposite trend to C–O and Cu–O (lattice), and the increase in EF strength shortens the C–O (lattice) lengths until *E* = 0.75 V/Å. Figure 3b illustrates the effects of EF on the desorption energy of CO_2_ on the CuO (111) surface with an O vacancy. The rising EF decreases the desorption energy of CO_2_, favoring the departure of adsorbed CO_2_ from the CuO (111) surface.

The effects of EF on CO_2_ desorption behaviors from the CuO (111) surface with an O vacancy at the electronic level were further explored. Bader charge and PDOS analyses were performed for the CO_2_ desorption process, respectively, as shown in Table 2. For C and O (lattice) atoms, the total C loss charge and O (lattice) obtained charge fluctuate in *E* = −1 to 1 V/ Å. When *E* ranges from −1 to 1 V/Å, the EF enhances the charge transfers from C to O. The EF inhibits electron transfers from O (lattice) to Cu and C to O (lattice). Based on the analysis in Section 3.1, O (lattice)&C and Cu atoms are in saturated and unsaturated states, respectively. Thus, the enhancement by rising EF for charge transfers from C to O and Cu to O (lattice) decreases the stability of C–O and Cu–O (lattice), and the corresponding bond length increase. By contract, the bond length of C–O (lattice) shows a downward trend with the increase of EF strength because of the inhibition influence of EF on electron transfers from C to O (lattice). The change in bond lengths is inconsistent with that of charge transfer in the case of *E* = 1 V/Å. When *E* is 1 V/Å, the changes in electron transfer for C → O, C → O (lattice), and O (lattice) → Cu are significantly higher than other conditions (over 10 times). The electrons transfer from C to O and O to Cu rise greatly and increase the shared electrons between atoms. More shared electrons could enhance the bond stability and shorten bond lengths. Similarly, in the *E* = 1 V/Å case, the bond length of C–O (lattice) decreases because of the reduction of shared electrons between C and O (lattice) atoms.

**Table 2:** Charge Transfers and Band Centers of Cu, O and C Atoms during CO_2_ Adsorbs on the CuO (111) Surface with an O Vacancy with *E* Ranging from −1 to 1 V/Å

**E**(V/Å)	**–1**	**–0.75**	**–0.5**	**–0.25**	**0**	**0.25**	**0.5**	**0.75**	**1**
Charge transfer (|*e*|)									
C → O	0.951	0.979	0.979	0.996	1.024	1.024	1.051	1.063	1.331
C → O (lattice)	1.177	1.164	1.131	1.104	1.118	1.082	1.050	1.040	0.773
O (lattice) → Cu	0.057	0.045	0.034	0.021	0.014	–0.001	–0.013	–0.025	–0.293
Total C loss charge	2.127	2.143	2.110	2. 100	2.142	2.106	2.101	2.103	2.105
Total O (lattice) obtain charge	1.120	1.118	1.097	1.083	1.104	1.083	1.063	1.065	1.067
**Bond center (eV)**									
Cu-3d	–2.560	–2.574	–2.626	–2.661	–2.675	–2.702	–2.702	–2.735	–2.738
O (lattice)-2p	–6.208	–6.544	–6.816	–7.131	–7.449	–7.755	–7.994	–8.280	–8.166

To further understand the effects of EF on CO_2_ desorption behaviors from the CuO (111) surface with an O vacancy at the electronic level, Bader charge and PDOS analysis were performed for the CO_2_ desorption process, respectively, as shown in Table2. For C and O (lattice) atoms, the total C loss charge and O (lattice) obtained charge fluctuate in *E* = −1 to 1 V/ Å. When *E* ranges from −1 to 1 V/Å, EF enhances the charge transfers from C to O, but it inhibits electron transfers from the O (lattice) to Cu and C to the O (lattice). Based on the analysis in Section 3.1, O (lattice)&C and Cu atoms are in saturated and unsaturated states, respectively. Thus, the enhancement by rising EF for charge transfers from C to O and Cu to O (lattice) decreases the stability of C–O and Cu–O (lattice), and the bond length of them will also increase correspondingly. By contract, the bond length of C–O (lattice) shows a downward trend with the increase of EF strength because of the inhibition influence of EF on electron transfers from C to O (lattice). However, the change in bond lengths is inconsistent with that of charge transfer in the case of *E* = 1 V/Å. When *E* is 1 V/Å, the changes in electron transfer for C → O, C → O (lattice) and O (lattice) → Cu are significantly higher than other conditions (over 10 times). A sharp variation in the shared electrons between atoms plays a key role in the bond lengths. Specifically, the electrons transferred from C to O and from O to Cu rise greatly and also increase the shared electrons between atoms. More shared electrons could enhance the bond stability and shorten the bond lengths. Similarly, in the *E* = 1 V/Å case, the bond length of C–O (lattice) decreases because of the reduction of shared electrons between C and O (lattice) atoms.

PDOS analysis was conducted for Cu-3d and the O (lattice)-2p orbits to better reveal the EF influence on the CO_2_ desorption energy. As illustrated in Table 2, both the Cu-3d and the O (lattice)-2p bond centers present a downward trend with *E* ranging from −1 to 0.75 V/Å, indicating that the interactions between Cu and the O (lattice) are weakened by rising EF strength. Thus, the desorption abilities of CO_2_ from the CuO surface become stronger, and the desorption energy decreases. When *E* is 1 V/Å, the Cu-3d bond center continues to move away from the Fermi energy level (0 eV); however, the bond center of the O (lattice)-2p bond increases. Considering the decrease in desorption energy in *E* = 1 V/Å, it can be concluded that the promotion influence of the Cu-3d bond center desorption plays a dominant role in CO_2_.

### 3.3 Effects of Electric Field on the Reactions of CO on CuO (111) Surface

**Figure 4:**
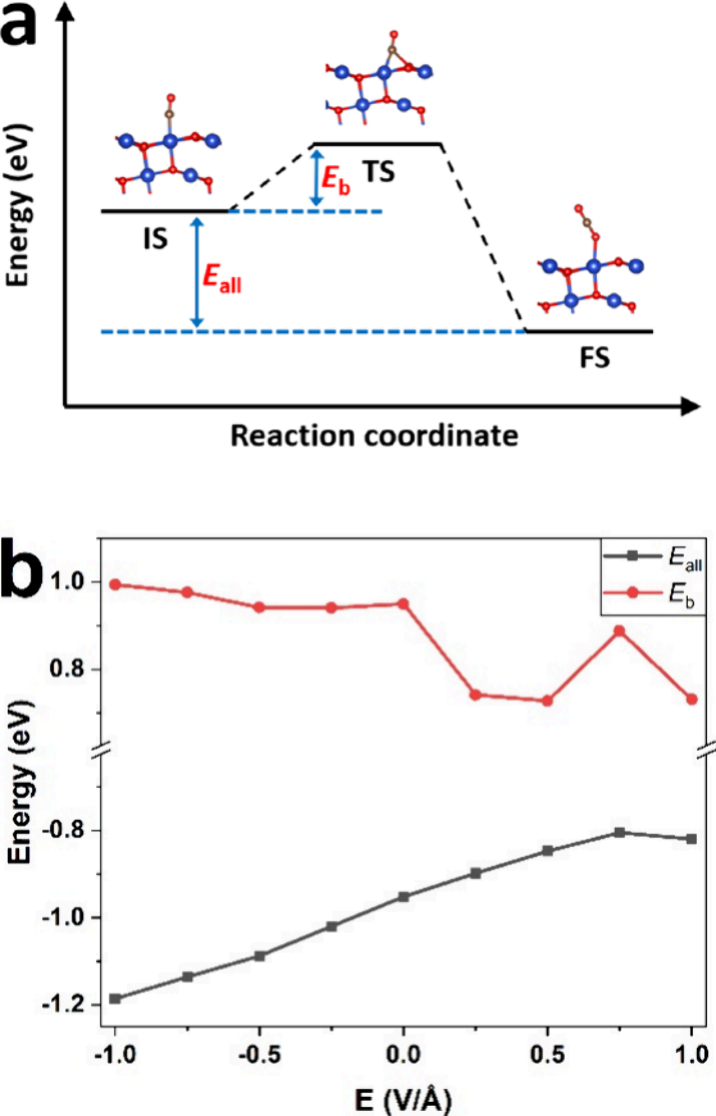
(a) Energy profile and structures of the conversion from CO to CO_2_ on the CuO (111) surface without EF applied via the MvK mechanism. Cu and O atoms are represented in blue and red, respectively. (b) The influence of EF on the overall reaction energy (*E*_all_) and energy barrier (*E*_b_) during CO oxidation on the CuO (111) surface.

According to the MvK mechanism, adsorbed CO will react with lattice O to form adsorbed CO_2_ on the CuO (111) surface with an O vacancy. In this section, the chemical process from CO to CO_2_ on the CuO (111) surface is investigated in the *E* = 0 case. Figure 4a shows the energy profile and structures of the conversion from CO to CO_2_ on the CuO (111) surface following the MvK mechanism. The absorbed CO and CO_2_ are the initial state (IS) and final state (FS) of the reaction process, respectively. A transition state (TS) between the IS and FS is a critical state with the highest energy. Energy barrier (*E*_b_) and the energy difference between TS and IS, is the key indicator for reaction rates. *E*_all_ represents the total energy change during the reaction, which is the energy difference between the FS and IS. *E*_b_ and *E*_all_ are 0.950 eV and −0.952 eV, respectively, under EF-free conditions.

The overall reaction energy (*E*_all_) and energy barrier (*E*_b_) in the CO combustion on the CuO (111) surface under different EF strengths are shown in Figure 4b. *E*_all_ increases from −1.186 to −0.805 eV with EF strength ranging from −1 to 0.75 V/Å, which means that the rising EF inhibits heat release in the CO oxidation on the CuO surface. EF promotes heat release in the CO combustion in the *E* = 1 V/Å case. Regarding reaction barriers, positive EF has a greater influence on *E*_b_ than negative EF. Specifically, when EF strength is larger than 0 V/Å, *E*_b_ presents a parabolic trend and reaches its lowest point, 0.727 eV, with an *E* of 0.5 V/Å. In the *E* = −1 to 0 V/Å cases, *E*_b_ first decreases until *E* = −0.25 V/Å with the increase in EF strength. The EF can reduce the energy barrier during CO oxidation on the CuO surface by about 23% and accelerate the CO combustion, correspondingly.

## 4 Discussion

In this study, we systematically explored the behaviors of CO oxidation on the CuO (111) surface with and without an EF. The effects of EF on CO adsorption, overall reaction energy, energy barrier, and CO_2_ desorption were investigated and analyzed during CO combustion on the CuO (111) surface.

According to the DFT results, both the magnitude and direction of the EF have significant effects on the oxidation characteristics of CO on the CuO (111) surface. EF along the −Z direction can significantly benefit CO adsorption and overall reaction energy; it slightly lowers the reaction energy barrier, however, inhibiting CO_2_ desorption. For EF along the +Z direction, it can promote CO adsorption, CO_2_ desorption, and reaction rates by reducing energy barriers, but it hinders the exothermic heat of CO oxidation. Such findings demonstrate that the application of EF could be an effective method to mitigate sintering and agglomeration of CuO by decreasing operating temperatures with negligible loss of combustion efficiency. The choice of electric field strength and direction should be based on the practical requirements. Further evaluation of kinetic characteristics for CO adsorption, CO combustion, and CO_2_ desorption rates is required.

In the present study, the MvK mechanism is considered, and the influence of EF on other mechanisms such as ER and LH can be explored for future work. The application of EF to other OC materials and fuels may also be an effective way to improve the CLC performance, which warrants further investigation.

## 5 Conclusions

CO oxidation on the CuO (111) surface following the MvK mechanism was investigated under different EF strengths via DFT calculations. The EF influence on CO adsorption and CO_2_ desorption on the CuO (111) was clarified at the electron level for the first time. The effects of EF on the reaction barriers and overall reaction energy were revealed. Results indicate that both positive and negative EF can enhance the CO adsorption on the CuO (111) surface, and the negative EF presents a better promotion influence on CO adsorption than positive EF. For CO_2_ desorption, positive EF lowers the desorption energy of CO_2_ and benefits CO_2_ desorption from the CuO (111) surface. The negative and positive EFs present promotion and inhibition effects on heat release of CO oxidation on the CuO (111) surface, respectively. Finally, both negative and positive EF can lower the energy barrier for CO oxidation on the CuO (111) surface. Specifically, positive EF shows better performance on energy barrier reduction, which can decrease the energy barrier by 23%. This study demonstrates that the EF can promote the CLC process and can be used to control CLC behaviors. For future work, application of EF in other OC materials and fuels deserves further study, which can help expand the application area and improve performance of the CLC process.
